# Single-Cell Transcriptomics Reveals a Multi-Compartmental Cellular Cascade Underlying Elahere-Induced Ocular Toxicity in Rats

**DOI:** 10.3390/ph18101492

**Published:** 2025-10-04

**Authors:** Jialing Zhang, Meng Li, Yuxuan Yang, Peng Guo, Weiyu Li, Hongxin An, Yongfei Cui, Luyun Guo, Maoqin Duan, Ye Lu, Chuanfei Yu, Lan Wang

**Affiliations:** 1 State Key Laboratory of Drug Regulatory Science, NHC Key Laboratory of Research on Quality and Standardization of Biotech Products, NMPA Key Laboratory for Quality Research and Evaluation of Biological Products, National Institutes for Food and Drug Control, Beijing 102629, China; zhangjialing@nifdc.org.cn (J.Z.); limeng@nifdc.org.cn (M.L.); liweiyu@nifdc.org.cn (W.L.); ahx0304@163.com (H.A.); cuiyongfei@nifdc.org.cn (Y.C.); guoluyun@nifdc.org.cn (L.G.); duanmaoqin@nifdc.org.cn (M.D.); 2 Clinical and Translational Research Center, Hangzhou Institute of Medicine (HIM), Chinese Academy of Sciences, Hangzhou 311121, China; yxyang999@gmail.com (Y.Y.); guopeng@ucas.ac.cn (P.G.)

**Keywords:** antibody-drug conjugate (ADC), Elahere (Mirvetuximab Soravtansine), ocular toxicity, corneal toxicity, single-cell RNA sequencing

## Abstract

**Background:** Antibody-drug conjugates (ADCs) have ushered in a new era of precision oncology by combining the targeting specificity of monoclonal antibodies with the potent cytotoxicity of chemotherapeutic drugs. However, the cellular and molecular mechanisms underlying their dose-limiting ocular toxicity remain unclear. Elahere™, the first FDA-approved ADC targeting folate receptor α (FRα), demonstrates remarkable efficacy in platinum-resistant ovarian cancer but causes keratitis and other ocular toxicities in some patients. Notably, FRα is not expressed in the corneal epithelium—the primary site of damage—highlighting the urgent need to elucidate its underlying mechanisms. The aim of this study was to identify the cell-type-specific molecular mechanisms underlying Elahere-induced ocular toxicity. **Methods:** Sprague-Dawley rats were treated with intravenous Elahere (20 mg/kg) or vehicle weekly for five weeks. Ocular toxicity was determined by clinical examination and histopathology. Corneal single-cell suspensions were analyzed using the BD Rhapsody single-cell RNA sequencing (scRNA-seq) platform. Bioinformatic analyses to characterize changes in corneal cell populations, gene expression, and signaling pathways included cell clustering, differential gene expression, pseudotime trajectory inference, and cell-cell interaction modeling. **Results:** scRNA-seq profiling of 47,606 corneal cells revealed significant damage to the ocular surface and corneal epithelia in the Elahere group. Twenty distinct cell types were identified. Elahere depleted myeloid immune cells; in particular, homeostatic gene expression was suppressed in phagocytic macrophages. Progenitor populations (limbal stem cells and basal cells) accumulated (e.g., a ~2.6-fold expansion of limbal stem cells), while terminally differentiated cells decreased in corneal epithelium, indicating differentiation blockade. Endothelial cells exhibited signs of injury and inflammation, including reduced angiogenic subtypes and heightened stress responses. Folate receptor alpha, the target of Elahere, was expressed in endothelial and stromal cells, potentially driving stromal cells toward a pro-fibrotic phenotype. Fc receptor genes were predominantly expressed in myeloid cells, suggesting a potential mechanism underlying their depletion. **Conclusions:** Elahere induces complex, multi-compartmental ocular toxicity characterized by initial perturbations in vascular endothelial and immune cell populations followed by the arrest of epithelial differentiation and stromal remodeling. These findings reveal a cascade of cellular disruptions and provide mechanistic insights into mitigating Elahere-associated ocular side effects.

## 1. Introduction

Antibody-drug conjugates (ADCs), which combine the target specificity of monoclonal antibodies with the potent cell-killing efficacy of cytotoxic drugs, have heralded a new era in precision oncology [1,2,3,4]. The archetypal ADC structure includes three components: an antibody directed against a tumor-associated antigen, a linker molecule and a highly potent cytotoxic payload, such as maytansinoid derivatives or auristatins [5,6]. This design facilitates antigen-mediated endocytosis of ADCs by tumor cells followed by the intracellular release of toxins to selectively induce apoptosis [7,8]. Despite their clinical success in oncology, ADCs frequently exhibit dose-limiting adverse effects, particularly ocular toxicity, which remains poorly understood at the cellular and molecular levels in progenitor populations [9,10,11]. The unique anatomical and physiological features, including the blood-eye barrier and the high metabolic activity of corneal epithelial cells, render the eye susceptible to ADC-induced damage [12]. ADC-related ocular toxicity usually affects the epithelial cell layer, causing corneal damage. In contrast, endothelial cells, which are in contact with the anterior chamber aqueous humor, are usually unharmed by ADCs [13,14,15]. For example, trastuzumab deruxtecan is associated with corneal epitheliopathy and dry eye syndrome in approximately 30% of patients; this toxicity may be linked to *HER2* expression in corneal basal cells and non-specific drug permeation [16,17]. Thus, ocular toxicity is not solely due to off-target effects; tissue-specific antigen expression and cellular uptake mechanisms may also contribute to ocular toxicity. Elahere™ (mirvetuximab soravtansine), the first FDA-approved folate receptor alpha (FRα)-targeting ADC, is remarkably effective in patients with platinum-resistant ovarian cancer [18,19]. Elahere is a combination of humanized anti-FRα antibody and the maytansinoid derivative DM4, with a cleavable linker facilitating intracellular toxin release after target binding [20,21]. In phase III clinical trials, Elahere exhibited a good median progression-free survival of 5.6 months compared to chemotherapy in patients with platinum-resistant ovarian cancer [18]. However, real-world data highlight the significant incidence of ocular toxicity, affecting up to 12.3% of patients. The ocular toxicity induced by Elahere manifests as keratitis, blurred vision, and other corneal adverse events [22,23]. Intriguingly, corneal epithelial cells are the primary site of clinical damage, but FRα expression has not been detected in these cells, suggesting that toxicity may not be caused by direct on-target effects. Potential mechanisms for Elahere-induced ocular toxicity include secondary epithelial damage induced by FRα expression in other corneal compartments (e.g., endothelium, stroma, or immune cells), off-target effects, or payload-related toxicity independent of FRα binding. However, the molecular mechanisms underlying Elahere-induced ocular toxicity, including cell type-specific responses and disrupted signaling pathways, are unclear.

Traditional studies, such as histopathological analysis, do not completely capture the cellular heterogeneity and dynamic molecular changes induced by ADCs in complex tissues [24]. In contrast, single-cell RNA sequencing (scRNA-seq) enables the dissection of individual cellular transcriptomes within heterogeneous tissues [25,26,27]. In ophthalmology, scRNA-seq has been successfully applied to characterize the limbal niche in cynomolgus monkeys and uncover RNA splicing and expression patterns in corneal tissue [28]. In this study, scRNA-seq was used to systematically investigate Elahere-induced differential gene expressions and pathway activation (e.g., apoptosis, inflammation, and extracellular matrix remodeling) across diverse corneal cell types, including basal epithelial cells, immune cells, and stromal cells [29,30].

A rat model of Elahere-induced ocular toxicity was developed to compare corneal transcriptomic profiles in the presence or absence of Elahere using scRNA-seq. To our knowledge, this is the first study using scRNA-seq to investigate the ocular toxicity of an FRα-targeting ADC. The scRNA-seq results were integrated with in vivo observations to elucidate the cellular and molecular mechanisms of Elahere-induced ocular toxicity. These insights can provide the basis for developing next-generation ADCs with improved safety profiles and guide personalized dosing strategies, advancing translational medicine and precision oncology.

## 2. Results

### 2.1. Elahere Administration Induces Clinically and Histologically Evident Ocular Toxicity in Rats

**Figure 1:**
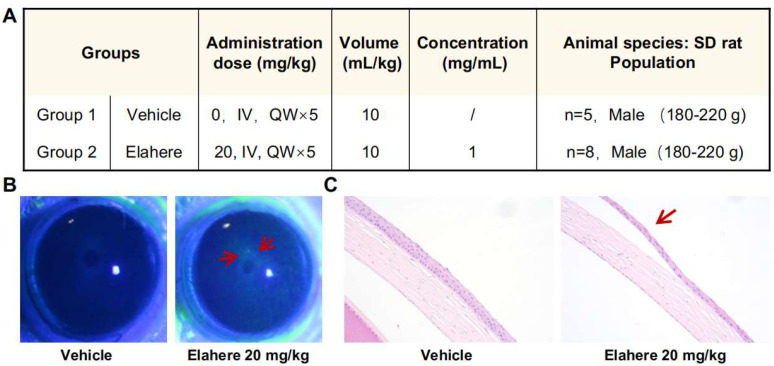
Elahere induces progressive ocular surface damage and corneal epithelial injury in rats. (**A**) Experimental design for the Elahere-induced ocular toxicity model, including the drug dose (mg/kg), volume (mL/kg), concentration (mg/mL), and animal species/population (SD rat, Vehicle n = 5, Elahere n = 8, Male 180–220 g, both groups treated QWx5). (**B**) Representative images of corneal fluorescein sodium staining in Vehicle- and Elahere-treated rats on day 21 of treatment. Arrows indicate areas of increased fluorescein uptake, signifying epithelial barrier disruption in the Elahere group. Scale bar = 1 mm. (**C**) Representative hematoxylin and eosin-stained corneal sections from Vehicle- and Elahere-treated rats at 5 weeks. Arrows in the Elahere group highlight corneal epithelial atrophy and cellular degeneration. Scale bar = 200 µm.

An immunocompetent rat model was used to evaluate the potential ocular toxicity of Elahere [31]. Male Sprague-Dawley rats were treated with intravenous Elahere (20 mg/kg) or vehicle (5% glucose solution) once weekly for five weeks (Figure 1A). A lower dose of Elahere (10 mg/kg for four weeks) induced only mild ocular changes; thus, a higher dose or longer duration was needed to elicit more pronounced toxicity suitable for mechanistic investigation. Fluorescein sodium staining revealed progressive corneal surface damage in the Elahere group. Fluorescein uptake increased significantly by day 21 in four of eight rats in the Elahere-treated group compared to the Vehicle-treated group, indicating corneal epithelial barrier disruption (Figure 1B). The fluorescein-stained regions appeared as distinct green patches, often concentrated in the central cornea, suggesting localized loss of epithelial integrity. Histopathological analysis of H&E-stained corneal sections after five weeks of treatment validated these findings. Vehicle-treated animals displayed normal corneal architecture. In contrast, three of the Elahere-treated rats showed prominent pathological changes in the epithelium, including epithelial atrophy, reduced epithelial cell layers, and evidence of cellular degeneration, such as nuclear pyknosis, karyorrhexis, and karyolysis (Figure 1C). Notably, the underlying stroma and endothelium appeared unaffected at the light microscopic level, indicating that the corneal epithelium was the primary site of injury.

Based on these results, a robust Elahere-induced ocular toxicity model was established in rats, characterized by clinically apparent and histologically confirmed corneal epithelial damage. This model provided the foundation for mechanistic studies using single-cell transcriptomics to investigate the molecular basis of Elahere-induced toxicity.

### 2.2. Single-Cell Transcriptomic Profiling Reveals Cellular Changes in the Cornea After Elahere Treatment

**Figure 2:**
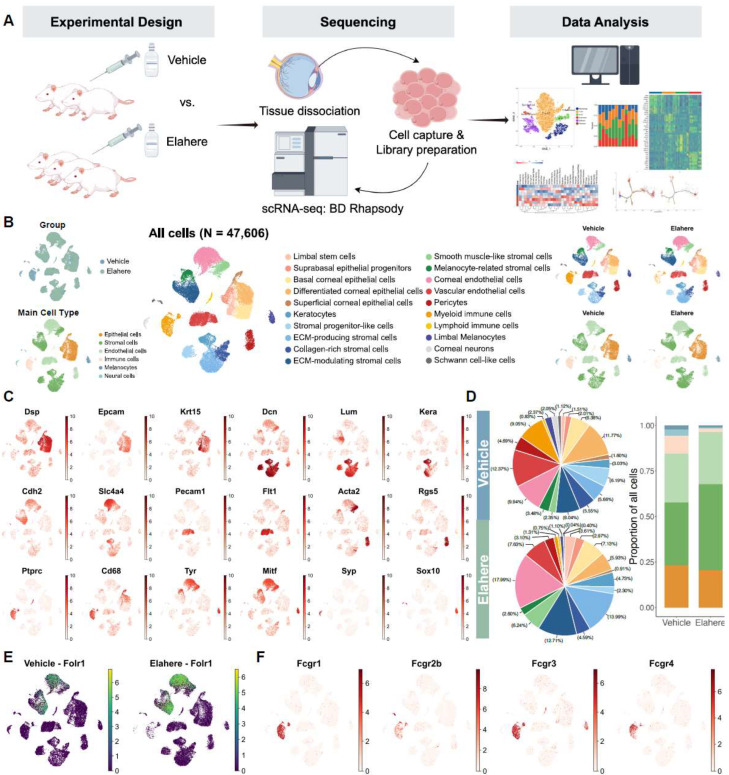
Single-cell transcriptomic landscape of the cornea reveals a heterogeneous response to Elahere treatment. (**A**) Overview of the experimental design and workflow for the scRNA-Seq analysis, depicting sample collection from Vehicle and Elahere-treated rats, corneal tissue processing, single-cell isolation, library preparation, sequencing, and bioinformatics analysis. (**B**) UMAP plot illustrating the clustering of 47,606 cells into 20 distinct cell clusters (center). The four corner insets display UMAP plots of the group distribution (top-left, Vehicle vs. Elahere), the annotated main cell types (bottom-left), and the cell-type distributions in the Vehicle and Elahere groups (top-right and bottom-right, respectively). (**C**) UMAP feature plots showing the expression patterns of selected canonical marker genes across all cells, emphasizing their specificity to individual cell types (e.g., *Dsp* and *Krt15*: epithelial cells; *Dcn* and *Kera*: stromal cells; *Pecam1* and *Cdh2*: endothelial cells; *Ptprc* and *Cd68*: immune cells; *Tyr*: melanocytes; and *Syp*: neural cells). (**D**) Cellular composition analysis. The pie charts summarize the proportional distribution of each major cell type in the Vehicle (top) and Elahere (bottom) groups. The bar plot (far right) depicts the fold changes in cell-type proportions in the Elahere group relative to the Vehicle group, revealing shifts in major cell populations (overall changes are statistically significant, *p* < 0.001, Chi-squared test). The colors are consistent with panel B (main cell types). (**E**) UMAP feature plot illustrating *Folr1* mRNA expression across all annotated corneal cell types. Folr1 levels were higher in the endothelial and some stromal clusters. (**F**) UMAP feature plots showing the expression of Fc gamma receptor genes (*Fcgr1*, *Fcgr2b*, *Fcgr3a*, and *Fcgr4*) in corneal cells, highlighting the predominant expression in myeloid cell clusters.

To investigate the cellular basis of Elahere-induced ocular toxicity, scRNA-seq analysis of corneal tissues, including the limbal region, from rats treated with Vehicle or Elahere was conducted (Figure 2A). After stringent quality control and filtering, 47,606 high-quality cells were retained for analysis, including 19,518 cells (41%) from Vehicle-treated animals and 28,088 cells (59%) from Elahere-treated animals. Twenty distinct cell clusters were identified using unsupervised clustering and UMAP visualization of the integrated dataset (Figure 2B, center; Supplementary Figure S1). Based on canonical marker gene expression, the cell clusters were derived from six major corneal cell types (Figure 2B, bottom-left inset; Figure 2C). The cell types included epithelial cells expressing *Dsp* and *Epcam*, with *Krt15* marking limbal stem cells; stromal cells expressing *Dcn* and *Lum* with *Kera* specific for keratocytes, endothelial cells, including corneal endothelium expressing *Cdh2* and *Slc4a4*, vascular endothelium expressing *Pecam1* and *Flt1*, and pericytes expressing *Acta2* and *Rgs5*; immune cell populations expressing *Ptprc* and *Cd68*; and smaller populations of melanocytes expressing *Tyr* and *Mitf* and neural cells expressing *Syp* and *Sox10* (Figure 2C). The distribution of these cells differed between the Vehicle and Elahere groups (Figure 2B, top-left, top-right, and bottom-right insets).

Elahere treatment led to substantial remodeling of the corneal cellular landscape, as indicated by significant shifts in cell type proportions (Figure 2D, *p* < 0.001 by chi-square test). Within the epithelial compartment, progenitor populations expanded (e.g., LSCs expanded ~2.6-fold), while the proportion of terminally differentiated superficial cells decreased by 35.7%. The stromal compartment also underwent significant changes, characterized by a severe depletion of progenitor-like stromal cells (from 22.2% to 5.2%) and a near-doubling of ECM-producing fibroblasts.

To explore the direct effects of Elahere on the cornea, the gene expression of its molecular target, folate receptor alpha (*Folr1*/FRα), in the different corneal cell populations was determined. *Folr1* mRNA was predominantly expressed in corneal endothelial cell and stromal-like cell subclusters (Figure 2E). Notably, *Folr1* expression appeared more pronounced in Elahere-treated samples. Consistent with previous reports, FRα expression in epithelial cells was low to absent. This expression pattern indicates that if toxicity is mediated by on-target FRα binding, endothelial and stromal cells are the primary direct targets, not the corneal epithelium, where the most prominent clinical damage was detected.

Elahere is an antibody-drug conjugate, which includes an Fc region capable of interacting with Fc receptors. Thus, the expression of Fc gamma receptor (FcγR) genes (*Fcgr1*, *Fcgr2b*, *Fcgr3a*, and *Fcgr4*) was investigated. Fc receptor genes were highly and selectively expressed in myeloid cell clusters, particularly macrophages (Figure 2F). Fc receptors were minimally expressed in lymphoid cells and virtually absent in non-immune corneal cells, including epithelial, stromal, and endothelial cells. This selective expression pattern suggests that macrophages are particularly vulnerable to Elahere-induced Fc-mediated effects, such as antibody-dependent cellular cytotoxicity (ADCC), antibody-dependent cellular phagocytosis (ADCP), or other FcγR-mediated clearance mechanisms. The Fcgr expression pattern explains the depletion of myeloid cells, especially macrophages, following Elahere treatment.

Overall, these findings suggest a multi-pronged impact of Elahere, involving direct effects on FRα-expressing endothelial and stromal cells and Fc-mediated effects on myeloid cells. The scRNA-seq results highlight a complex cellular response to Elahere, involving shifts in epithelial differentiation status, profound immune cell loss, and stromal activation.

### 2.3. Elahere Induces Immune Cell Depletion and Reprogramming, Especially in Macrophages

**Figure 3:**
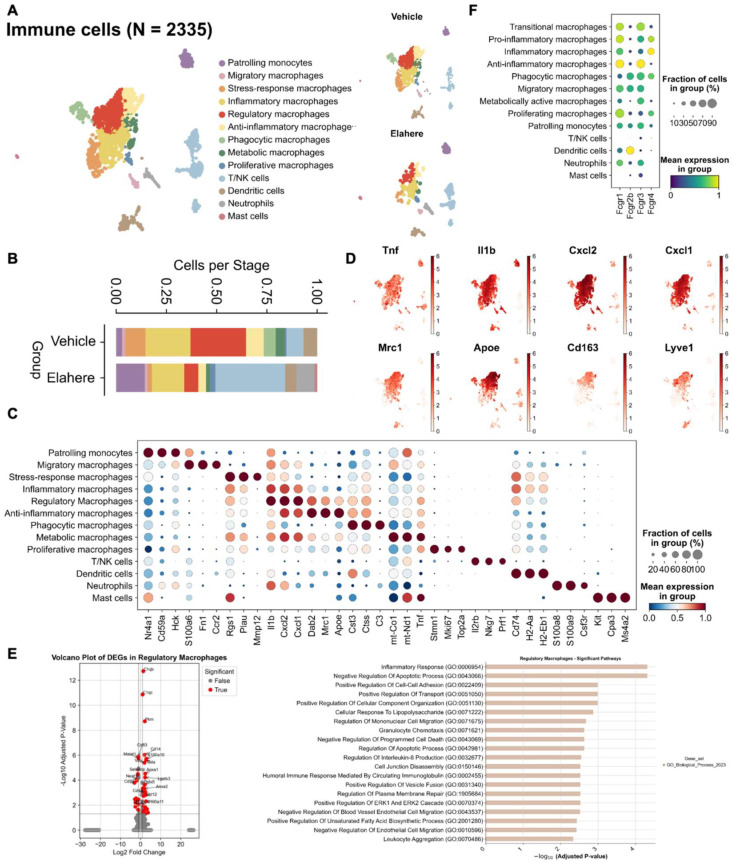
Elahere treatment depletes and reprograms corneal macrophages. (**A**) UMAP visualization of 2335 re-clustered immune cells from Vehicle- and Elahere-treated corneas, showing 13 distinct subsets, including diverse macrophage populations (e.g., phagocytic macrophages and pro-inflammatory macrophages), monocytes, dendritic cells, neutrophils, mast cells, and T/NK cells. (**B**) Stacked bar plots illustrating the relative proportions of the immune cell subsets in the Vehicle and Elahere groups. (**C**) Dot plot showing the expression levels (color intensity) and percentages of cells expressing (dot size) selected marker genes for each identified immune cell cluster. (**D**) UMAP feature plots depicting the average expression of representative pro-inflammatory genes (e.g., *Tnf*, *Il1b*, *Cxcl1*, and *Cxcl2*) and anti-inflammatory genes (e.g., *Mrc1*, *ApoE*, and *Cd163*) within macrophage subsets. (**E**) Violin plots showing normalized expression of differentially expressed genes between the Vehicle and Elahere groups and the enriched pathways. (**F**) UMAP feature plots showing the expression of FcR genes in the re-clustered immune cell populations, confirming the high expression in macrophage subsets.

Elahere treatment significantly altered the immune cell compartment of the cornea. Re-clustering of 2335 immune cells based on well-known markers resulted in 13 distinct subsets, including monocytes, macrophages (migratory, stress-response, inflammatory, regulatory, anti-inflammatory, phagocytic, and metabolic macrophages), T/NK cells, dendritic cells, neutrophils, and mast cells (Figure 3A,C). Macrophages, the predominant immune cell type in Vehicle-treated corneas, decreased significantly in response to Elahere treatment (Figure 3B). Phagocytic macrophages, crucial for debris clearance and tissue homeostasis, were almost completely absent in the Elahere group, representing a 100% reduction from their baseline proportion within the immune cell population. Conversely, the proportion of T/NK cells slightly increased, possibly due to the relative resistance of T/NK cells to Elahere-induced depletion or a consequence of the substantial loss of other immune cell types (Figure 3B). The macrophage populations in the Elahere-treated group were enriched in pro-inflammatory markers (e.g., *Tnf*, *Il1b*, *Cxcl1*, and *Cxcl2*) compared to anti-inflammatory markers (*Mrc1*, *Apoe*, *Cd163*, and *Lyve1*) (Figure 3D).

The surviving macrophages displayed significant functional reprogramming. Macrophages (pro-inflammatory, anti-inflammatory, and regulatory subpopulations) partially retained inflammation-related functions after Elahere treatment, but chemotaxis, apoptosis regulation, and metabolic functions were significantly affected (Figure 3E, Supplementary Figure S2). Pro-inflammatory macrophages partially retained their inflammation-related functions after Elahere treatment, but their clearance capacity and metabolic activity were altered by Elahere. Anti-inflammatory macrophages tended to maintain inflammatory balance after Elahere treatment, but lipid metabolism and antioxidant functions were significantly regulated. The complement gene *C1qb* was significantly upregulated in inflammation-related macrophage populations, possibly reflecting altered complement pathway activity. *Fcgr1* and *Fcgr3* were highly expressed in macrophage subpopulations, including those with anti-inflammatory characteristics (Figure 3F); thus, the effects of Elahere on macrophages are likely FcγR-mediated (as suggested by Figure 2F). The dual impact of Elahere on macrophages, including extensive depletion and functional reprogramming of the survivors, may be crucial to the adverse effects of Elahere in the cornea.

### 2.4. Elahere Disrupts Corneal Epithelial Differentiation and Promotes Progenitor Accumulation

**Figure 4:**
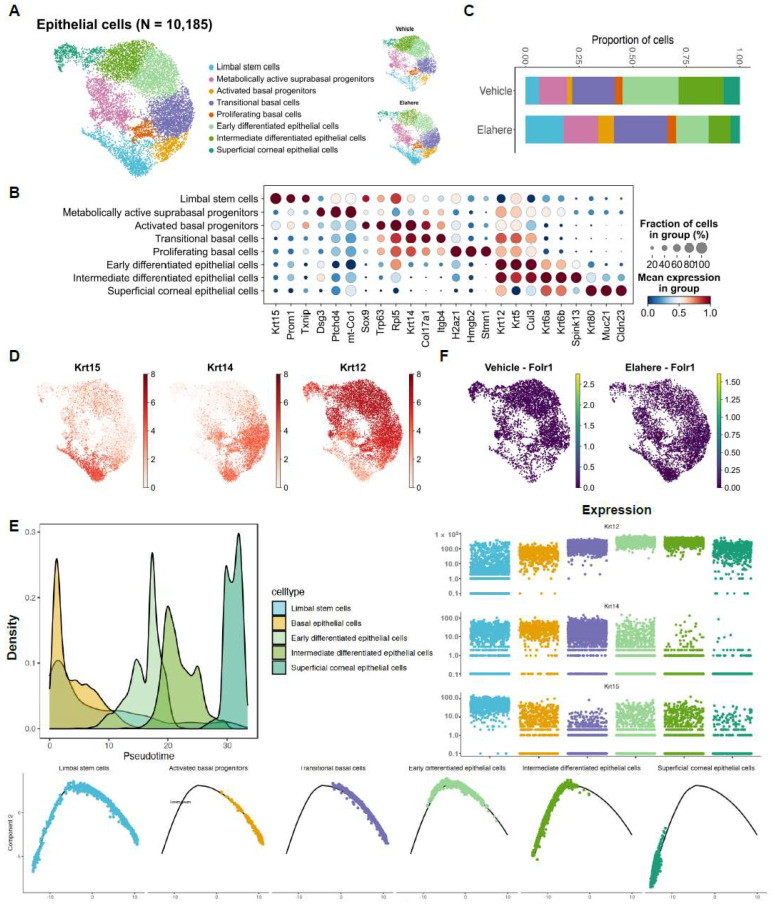
Elahere disrupts corneal epithelial differentiation and promotes progenitor cell accumulation. (**A**) UMAP plot of 10,185 re-clustered corneal epithelial cells from Vehicle- and Elahere-treated rat corneas. Eight cell subsets, ranging from limbal stem cells (LSCs) to terminally differentiated superficial cells, were identified. Cells are colored by cluster, with separate UMAPs for Vehicle and Elahere conditions. (**B**) Dot plot showing the expression level (color intensity) and percentage of cells expressing (dot size) key marker genes for each identified epithelial cell subset. (**C**) Stacked bar plots showing the relative proportions of the epithelial cell subsets in the Vehicle and Elahere groups. (**D**) UMAP feature plots displaying the expression of key epithelial differentiation markers: *Krt12*, *Krt14*, and *Krt15*. (**E**) Pseudotime trajectory analysis of epithelial cells, with LSCs defined as the root (origin). The top right panel illustrates the dynamic expression of *Krt15*, *Krt14*, and *Krt12* along the pseudotime trajectory. The bottom panels show cell density distribution along the trajectory in the Vehicle and Elahere groups, demonstrating an accumulation of cells in earlier differentiation states in the Elahere group. (**F**) UMAP feature plots showing low to absent expression of Folr1 in epithelial cells from both Vehicle and Elahere groups.

Elahere induced profound disturbances in the differentiation hierarchy of the corneal epithelium, which is essential for maintaining ocular surface integrity and vision. Re-clustering of 10,185 epithelial cells revealed eight distinct cell subsets representing a differentiation continuum from limbal stem cells (LSCs) to terminally differentiated superficial cells (Figure 4A). These subsets were annotated using canonical markers, such as high *Krt15* in LSCs, *Krt14* in basal cells, and *Krt12* in superficial cells (Figure 4B,D). Following Elahere treatment, the cellular composition of the epithelium shifted dramatically. Progenitor populations, including LSCs and basal cells, expanded from ~45% of the epithelium in the vehicle group to ~70% in the Elahere group. Conversely, terminally differentiated superficial cells were significantly depleted, with their proportion among all epithelial cells decreasing by 35.7% (Figure 4C). This suggests a robust proliferative response combined with an arrest in differentiation.

To investigate this differentiation blockade, pseudotime trajectory analysis was performed, defining LSCs (expressing *Krt15*) as the origin of the differentiation path. This analysis provided further evidence supporting a disruption in the epithelial differentiation hierarchy (Figure 4E, top left panel shows the combined trajectory; bottom panels show cell densities for the Vehicle and Elahere groups along the trajectory). In the Elahere group, cells were enriched at earlier pseudotime points, whereas cells from the Vehicle group progressed further along the differentiation trajectory, indicating that the normal progression towards terminal differentiation was impaired following Elahere treatment. The dynamic expression of key keratin markers along this trajectory corroborated this conclusion (Figure 4E, top right panel). Specifically, *Krt15* (LSC/basal marker) expression was prominent in early-stage cells, while the progression to high *Krt14* (basal/suprabasal marker) and subsequently *Krt12* (terminal differentiation marker) expression was diminished in the Elahere group compared to the Vehicle group. Overall, these results suggest that Elahere may indirectly disrupt corneal epithelial homeostasis, likely due to alterations in the corneal microenvironment induced by its effects on other cell types, rather than by directly targeting the epithelium (consistent with low *Folr1* expression in epithelial cells, Figure 2E and Figure 4F). The epithelium appears to initiate a regenerative response by expanding its progenitor pool; however, these cells fail to terminally differentiate, leading to impaired barrier function and, eventually, keratopathy. This “failed regeneration” is a critical aspect of the observed toxicity.

### 2.5. Elahere Triggers Endothelial Cell Injury and Pro-Fibrotic Stromal Remodeling

**Figure 5:**
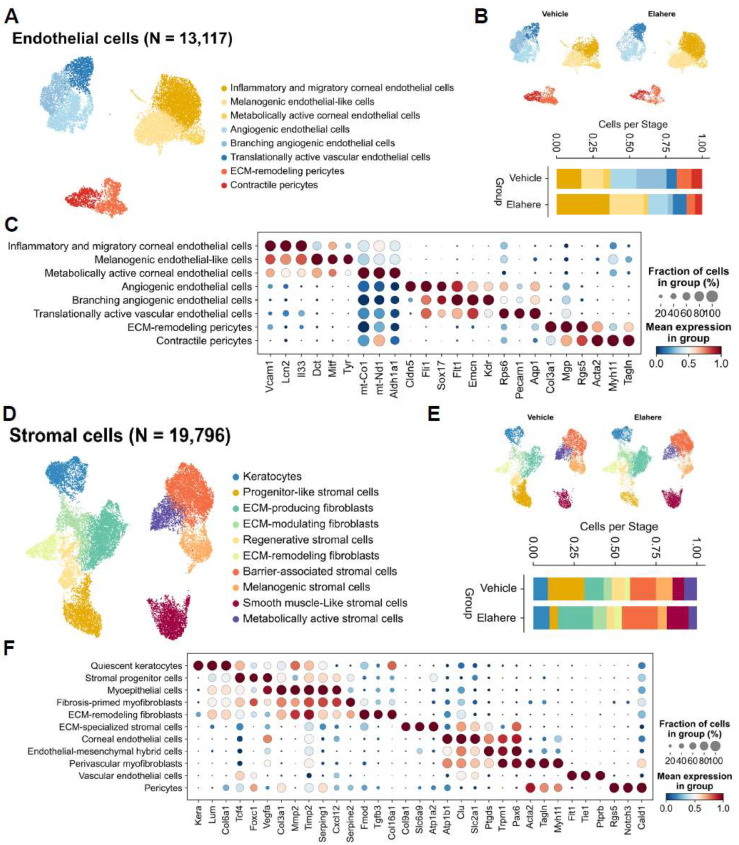
Elahere causes endothelial cell injury and pro-fibrotic stromal remodeling. (**A**) UMAP plot showing the re-clustering of 13,117 corneal endothelial cells from the Vehicle- and Elahere-treated groups into 8 subtypes. (**B**) Stacked bar plots showing the fractional proportions of each endothelial cell subtype in the Vehicle and Elahere groups. (**C**) Dot plot showing the expression of marker genes across the endothelial cell subtypes. The dot size and color intensity represent the fraction of cells in the subtype expressing the gene and the mean expression level, respectively. The genes include markers for inflammation (*Vcam1*, *Lcn2*), endothelial identity (*Pecam1*, *Cdh5*), angiogenesis (*Kdr*, *Flt1*), and pericyte markers (*Rgs5*, *Acta2*). (**D**) UMAP visualization of 19,796 re-clustered corneal stromal cells annotated into 10 subtypes, including keratocytes, progenitor-like stromal cells, ECM-producing/modulating/remodeling fibroblasts, and smooth muscle-like stromal cells. (**E**) Stacked bar plots showing the fractional proportion of each stromal cell subtype in the Vehicle (top) and Elahere (bottom) groups. (**F**) Dot plot showing the expression of marker genes, including keratocyte markers (*Lum*, *Kera*), ECM components (*Col1a1*), fibrosis markers (*Acta2*, *Fn1*), and progenitor cell markers, across the stromal cell subtypes. The dot size and color intensity represent the fraction of cells in the subtype expressing the gene and the mean expression level, respectively.

In addition to the immune and epithelial compartments, Elahere treatment induced substantial changes in the corneal endothelial and stromal niches, highlighting the multi-compartmental nature of Elahere toxicity. Endothelial cells (n = 13,117) were re-clustered into eight distinct subtypes, including inflammatory and migratory corneal endothelial cells, angiogenic endothelial cells, and pericyte populations (Figure 5A). Elahere treatment shifted the proportions of these endothelial cell subtypes. Specifically, the proportion of branching angiogenic endothelial cells was drastically reduced (from 20.6% to 3.3%), while inflammatory and migratory corneal endothelial cells more than doubled (from 17.0% to 35.2%) (Figure 5B). Dot plot analysis of marker gene expression revealed the upregulation of genes in inflammatory endothelial cells, including *Vcam1* and *Lcn2*, and the downregulation of genes associated with mature vasculature and quiescence, including *Pecam1* and *Cdh5*, in specific angiogenic clusters in the Elahere group (Figure 5C). Based on *Folr1* expression in endothelial cells (Figure 2E), these changes, characterized by a loss of angiogenic potential and a gain of inflammatory features, likely represent the direct on-target effects of Elahere. These changes contribute to vascular dysfunction and induce a pro-inflammatory microenvironment.

Stromal cells (n = 19,796) were reclustered into 10 distinct cell sub-types, including keratocytes, progenitor-like stromal cells, various fibroblast populations (ECM-producing, ECM-modulating, ECM-remodeling), and smooth muscle-like stromal cells (Figure 5D). Progenitor-like stromal cells were severely depleted (from 22.2% to 5.2%), while ECM-producing fibroblasts (from 11.8% to 21.4%) and smooth muscle-like stromal cells (from 7.0% to 13.3%) nearly doubled in proportion (Figure 5E). Gene expression analysis (Figure 5F) revealed that the expression of ECM components (e.g., *Col1a1*) and fibrosis-associated genes (e.g., *Acta2*) increased in the activated fibroblast populations, and quiescent keratocyte markers (*Kera* and *Lum*) were prominent in their respective clusters in the Elahere group. The overall balance shifted from the quiescent state towards more active, fibrotic phenotypes, indicating a pro-fibrotic stromal response characterized by increased ECM deposition and remodeling that may contribute to corneal opacity and impaired function.

These results demonstrate that Elahere induces injury and activation in endothelial and stromal cells. The endothelial damage, which is likely initiated by on-target FRα effects, may compromise vascular integrity and contribute to inflammation. The stromal response, characterized by a shift towards fibrotic activity, represents a maladaptive repair process that may contribute to long-term corneal pathology.

### 2.6. Elahere Treatment Remodels Intercellular Communication Networks in the Cornea

**Figure 6:**
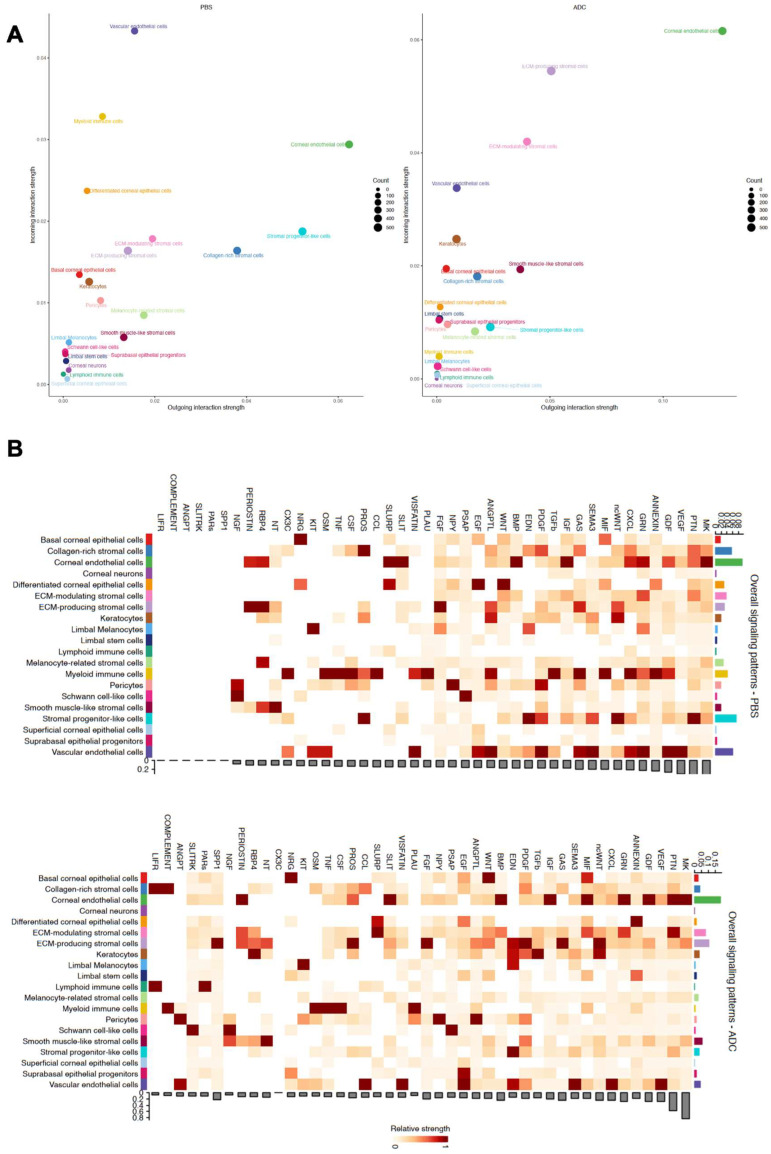
Elahere treatment remodels intercellular communication networks in the cornea. (**A**) Scatter plots illustrating the overall outgoing interaction strength (x-axis) versus the incoming interaction strength (y-axis) for each corneal cell type in the Vehicle (PBS, left plot) and Elahere (ADC, right plot) groups. Each dot represents a color-coded cell type. Dot size corresponds to the counts (e.g., number of interactions or relative contribution). (**B**) Heatmaps displaying the relative strengths of communication pathways (columns) originating from various sender cell types (rows) in the Vehicle (top) and Elahere (bottom) groups. Color intensity represents the strength of signaling. The bar plot below each heatmap summarizes the total outgoing signaling strength for each pathway for all sender cells. The bar plot to the right of each heatmap summarizes the total outgoing signaling strength for each sender cell type for all pathways.

To understand how the cellular changes induced by Elahere are coordinated and propagated across different corneal compartments, intercellular communication networks were analyzed using CellChat. This analysis revealed a dramatic remodeling of signaling interactions in the various corneal cell types in response to Elahere treatment. The scatter plots of outgoing versus incoming interaction strength (Figure 6A) demonstrate a clear shift in the distribution of signaling activity for different cell types between the Vehicle (PBS, left plot) and Elahere (ADC, right plot) conditions. For instance, myeloid immune cells, which are prominent signalers in the Vehicle group with high outgoing and incoming interaction strengths, showed markedly reduced strengths in the Elahere group, consistent with their depletion. Conversely, cell types such as ECM-producing stromal cells and corneal endothelial cells appeared to adopt more significant signaling roles, particularly as senders of signals, in the Elahere-treated corneas.

A global overview of signaling pathway activity (Figure 6B) revealed distinct patterns in the Vehicle (top heatmap) and Elahere (bottom heatmap) groups. The heatmaps depict the relative strengths of various signaling pathways (columns) originating from different sender cell types (rows), with corresponding bar plots summarizing the total outgoing signaling strength per pathway (bottom of each heatmap) and per cell type (right of each heatmap). Several pathways crucial for tissue remodeling and inflammation were upregulated in the Elahere-treated group. For example, TGFβ signaling showed substantially increased strength, with Keratocytes and ECM-producing stromal cells emerging as major sender populations in the Elahere group. Corneal endothelial cells and collagen-rich stromal cells also contributed to this pathway. Similarly, fibronectin (*FN1*) and collagen signaling, critical for extracellular matrix dynamics, were enhanced, primarily originating from these activated stromal (including Keratocytes and ECM-producing stromal cells) and endothelial sources. The *SPP1* (Osteopontin) and VEGF signaling pathways also exhibited increased activity in the Elahere group, largely driven by these stromal and endothelial cell populations.

Conversely, and strikingly visualized by the reduced intensity across their respective row in the heatmap and the diminished height in the right-side bar plot, the sum of outgoing signals from myeloid immune cells across most pathways was drastically reduced in the Elahere group. This profound reduction reflects their depletion and functional alteration and included diminished contributions to pathways such as COMPLEMENT, TNF, CCL, and CXCL. The overall activity of antigen presentation pathways, MHC-I and MHC-II, also shifted; MHC-II signaling, predominantly from myeloid cells in the Vehicle group, was substantially decreased in the Elahere group due to myeloid cell loss. MHC-I signaling, which is broader, showed a more complex pattern of alteration across various cell types. Chemotactic signaling, such as the CXCL pathway, also saw a significant reduction in myeloid-derived signals, though some stromal and endothelial contributions persisted or were modified. These changes collectively suggest a complex rewiring of the corneal intercellular communication network by Elahere. Heightened pro-fibrotic (TGFβ, *FN1*, COLLAGEN), pro-inflammatory, and potentially pro-angiogenic (VEGF, *SPP1*) signaling from stromal cells (notably Keratocytes and ECM-producing stromal cells) and endothelial cells, coupled with reduced homeostatic or regulatory signaling from depleted immune cells, likely creates a microenvironment that drives the pathological changes, including epithelial dysfunction and stromal fibrosis, induced by Elahere.

## 3. Discussion

Ocular toxicity is a common and often dose-limiting adverse effect associated with ADCs. However, the precise mechanisms underpinning these toxicities in the complex microenvironment of the cornea are poorly understood. Clinical studies reported various corneal changes, including keratitis, epithelial thinning, and microcyst formation, in patients treated with ADCs such as belantamab mafodotin (anti-BCMA), tisotumab vedotin (anti-TF), and Elahere (anti-FRα) [13]. However, conventional histological and clinical assessments provide limited resolution in dissecting the intricate interplay of diverse corneal cell types during injury and response processes. In this study, high-resolution scRNA-seq was leveraged to comprehensively map Elahere-induced corneal toxicity in an immunocompetent rat model. Our results reveal a coordinated and multi-compartmental cellular cascade initiated by perturbations in vascular endothelial and immune cell populations that subsequently propagate to impair corneal epithelial differentiation and induce maladaptive stromal remodeling.

This study for the first time reveals that vascular endothelial injury is a key initiating event in the pathogenesis of corneal toxicity induced by Elahere. The scRNA-seq analysis revealed a significant depletion in branching angiogenic endothelial cells, which are vital for vascular maintenance and neovascularization processes, and an expansion of metabolically active and pro-inflammatory endothelial subtypes. Stress-response genes (e.g., *Hmox1* and *Nrf2*) and immune adhesion molecules (e.g., *Icam1*, *Ccl2*), indicative of endothelial activation, damage, and inflammation, were upregulated in the altered endothelial cells. Of note, *Folr1*, the target of Elahere, was detected in corneal endothelial cells, providing strong evidence for an on-target mechanism for ADC-induced vascular damage. Although bystander effects from payload diffusion or systemic exposure cannot be entirely excluded, the FRα expression pattern implies a direct interaction. The resultant vascular dysfunction leads to increased vascular permeability, compromised barrier integrity, and a pro-inflammatory microenvironment, which facilitate drug penetration into corneal tissues and promote the recruitment of inflammatory cells, to exacerbate the initial injury.

Elahere treatment also caused profound alterations within the corneal immune compartment. Myeloid cells, especially macrophages, were the most severely affected cell population. Phagocytic macrophages, which play essential roles in tissue homeostasis, repair, and cellular debris clearance, were completely depleted (a 100% reduction within the immune compartment) in the Elahere group compared to the Vehicle group. The functional profile of the surviving macrophages was significantly altered, including the suppression of genes associated with phagocytosis (*Mrc1*, *Cd163*, and *Stab1*) and metabolism. However, genes associated with pro-inflammatory cytokines (*Tnf*, *Il1b*, and *Cxcl1*) were retained. The high expression of Fc gamma receptor genes (*Fcgr1*, *Fcgr2b*, *Fcgr3a*, and *Fcgr4*) suggests that antibody-dependent mechanisms, such as ADCC, ADCP, or FcγR-mediated uptake of ADC-opsonized cells or debris, are responsible for the depletion of myeloid cells. This dual impact of Elahere on myeloid cells, including numerical depletion and functional dysregulation in the surviving cells, likely impairs immune surveillance, efficient debris clearance, and local tissue repair mechanisms, contributing to corneal damage.

The corneal epithelium exhibited profound disruption in homeostasis, characterized by a notable shift towards progenitor and stem-like states, despite the low to absent *Folr1* expression. LSCs expanded by approximately 2.6-fold, and the proportions of basal progenitor populations increased significantly. In contrast, terminally differentiated epithelial cells, including the superficial cells that form the primary barrier, declined by approximately 35.7% in the Elahere group, but not in the Vehicle group. Pseudotime trajectory analysis corroborated these findings, suggesting a clear differentiation blockade; epithelial cells accumulated in early developmental states and failed to progress to terminal differentiation in the Elahere group. Given the lack of direct targeting via FRα, these epithelial effects may be secondary to altered microenvironmental cues emanating from the damaged endothelium and stroma and the perturbed immune compartments. The expansion of progenitor populations may be a compensatory regenerative response to ongoing injury; however, the concomitant differentiation arrest prevents the restoration of a functional epithelial barrier, leading to corneal surface instability and clinical manifestations of keratopathy.

Elahere also induced corneal stromal compartment remodeling, characterized by a severe depletion of progenitor-like stromal cells (from 22.2% to 5.2% of all stromal cells) and a concurrent expansion of ECM-producing fibroblasts (from 11.8% to 21.4%). The expression of genes associated with fibrosis (*Col1a1*, *Col3a1*), matrix regulation (Timp1, *Serpine2*), and contractility (*Acta2*, *Tagln*) increased in activated fibroblasts. The proportions of barrier-forming stromal cells, identified by *Cldn11* and *Cdh11* expression, also increased, potentially as a compensatory response to maintain structural integrity during epithelial and endothelial damage. This reactive stromal profile indicates a shift toward a microenvironment rich in extracellular matrix, which may lead to increased tissue stiffness, restricted normal regeneration, and, eventually, corneal haze or scarring.

Our results demonstrate a multi-compartmental response to Elahere, leading to corneal toxicity. Our data suggest a model where vascular endothelial injury, likely mediated by *Folr1* expression, may initiate a cascade of events, including immune cell depletion via FcγR-mediated mechanisms and macrophage dysfunction. These primary insults disrupt the corneal microenvironment, impairing epithelial differentiation and triggering maladaptive stromal remodeling. Together, these processes compromise corneal homeostasis and regeneration, resulting in the clinical and histopathological features of keratopathy.

Compared with similar studies, this research has made a new breakthrough in explaining the impact of ADCs on corneal epithelium. Although past studies have found that ADCs can cause changes in corneal epithelium, they have insufficient exploration of the mechanism of differentiation arrest [16]. To address this gap, our study is the first to map Elahere-induced ocular toxicity at single-cell resolution, a pivotal advance over prior ADC toxicity studies that relied on bulk histology or limited cell-type analyses. Unlike previous work focusing on isolated cell damage [32,33], our scRNA-seq data reveal a coordinated, multi-compartmental cascade-initiated by FRα-mediated vascular endothelial injury, followed by FRα-driven myeloid cell depletion, epithelial differentiation arrest, and stromal fibrosis. This identifies ocular toxicity as a dynamic disruption of tissue homeostasis rather than isolated cell-type impairment, offering a novel holistic framework absent in earlier research.

Regarding ADC localization and corneal damage, existing studies exclusively link ADC-related corneal injury to the epithelial layer [16,17]. In contrast, our findings show corneal endothelial cells (*Folr1*-expressing) as direct Elahere targets, with reduced angiogenic subtypes and heightened inflammation—uncovering a previously unrecognized endothelial role in toxicity initiation, which redefines ADC-mediated ocular tissue perturbation.

On limbal stem cells (LSCs), prior studies suggested potential LSC damage, and case reports linked ADC-induced keratopathy to limbal origins, but lacked mechanistic depth [34,35]. Our work extends this by demonstrating a ~2.6-fold LSC expansion alongside differentiation arrest (via pseudotime analysis): accumulated LSCs and basal progenitors fail to progress to terminally differentiated superficial cells, causing “ineffective regeneration” of the epithelial barrier. This mechanistic detail fills a critical gap in understanding LSC-driven ADC toxicity.

This study provides a high-resolution cellular map of Elahere-induced ocular toxicity, offering insights into the vulnerabilities of various corneal cell types with translational implications. For example, corticosteroid eye drops are commonly used to manage ADC-induced ocular side effects [22]. However, corticosteroids primarily target inflammation and may not address the underlying vascular or epithelial dysfunction. Interventions aimed at stabilizing endothelial cells, protecting LSC niches, and modulating macrophage responses could complement existing treatments. Additionally, re-engineering the Fc domain of ADCs to reduce FcγR interactions may mitigate immune cell depletion and the associated toxicities.

Our findings provide actionable insights for clinical management of ADC-induced ocular toxicity. Several of the pathways identified in this study are already pharmacologically tractable. For example, heightened TGFβ-driven fibrosis in the stromal compartment suggests that anti-fibrotic interventions targeting TGFβ signaling could mitigate maladaptive extracellular matrix remodeling and preserve corneal clarity [36]. Similarly, upregulated VEGF signaling in activated endothelial cells aligns with the established use of anti-VEGF therapies in ophthalmology, which may help reduce inflammation-associated vascular leakage in this context [37]. Moreover, the selective enrichment of FcγR in corneal macrophages implicates Fc-mediated immune dysfunction, suggesting that transient, localized immunomodulation or prophylactic anti-inflammatory therapy could mitigate macrophage exhaustion. Collectively, these insights point to the possibility of repurposing established ophthalmologic agents such as anti-fibrotics, anti-VEGF drugs, and targeted immunomodulators as adjuncts to corticosteroid eye drops to provide a multi-pronged strategy for preserving ocular health during ADC therapy.

Beyond immediate clinical translation, our data also inform the rational design of next-generation ADCs with improved ocular safety. Reducing FcγR engagement through antibody backbone engineering (e.g., IgG4 isotypes, LALA/PGLALA mutations, or deglycosylated variants) may prevent corneal macrophage depletion. In addition, tumor-restricted activation platforms such as protease-activated or pH-sensitive masked antibodies and conditional linkers can help confine payload release to malignant tissues and minimize off-tumor exposure. Optimization of payloads and linkers toward intracellular activation with low membrane permeability could further reduce bystander toxicity in non-malignant ocular cells. Finally, integrating model-informed precision dosing with early biomarkers of ocular stress (e.g., endothelial activation or TGFβ pathway signatures) may guide personalized dosing strategies to balance efficacy and safety. Taken together, these additions provide a translational framework in which mechanistic insights not only suggest actionable adjunctive interventions for clinical practice but also inform the engineering of next-generation ADCs with safer therapeutic indices.

This study has several limitations. Species-specific differences in *Folr1* distribution, immune architecture, and corneal physiology may limit the direct translatability of our results to humans. As noted in prior studies on MIRV pathogenesis, immunohistochemistry detected no FRα expression in human corneal, limbal, or conjunctival tissues [13,38]. In contrast, our scRNA-seq data showed rat FRα is primarily expressed in corneal endothelial and stromal cells, with minimal expression in epithelial cells (Figure 2E), reflecting clear interspecies differences in FRα distribution across corneal compartments. We recognize this discrepancy may affect the direct translatability of our rodent model findings to humans, as Elahere’s on-target effects could vary by species due to differing FRα localization. To address this limitation, we aim to validate our conclusions through scRNA-seq analysis of human ocular tissues in future studies, to better bridge preclinical and clinical observations.

Additionally, the analysis was conducted at a single late time point (week 5), capturing established pathology but not early initiation events. The lack of temporal resolution limits understanding of initiation vs. progression events. Future studies employing a time-course design with earlier sampling points are needed to validate the causal relationships and determine the sequence of events between endothelial, immune, and epithelial changes. The role of corneal nerves, hinted at by the detection of neural lineages, also warrants further investigation. Corneal nerve dysfunction may contribute to pain and discomfort. Finally, the contribution of payload-related, non-specific toxicity cannot be entirely excluded. The DM4 payload, a potent microtubule-disrupting agent, could cause bystander toxicity to nearby cells if released into the microenvironment, a mechanism known to contribute to ocular surface changes from this class of agents.

## 4. Materials and Methods

### 4.1. Animal Model and Elahere Administration

Specific pathogen-free male Sprague-Dawley rats, aged 6–8 weeks, weighing 180–220 g, were used for this study. The animal study protocol was approved by the Institutional Animal Care and Use Committee (protocol: IACUC-SW-B2024133-E011-01; approval date: 23 July 2024). Elahere (Mirvetuximab Soravtansine, Lot p1947922001A, 100 mg/20 mL) was provided by ImmunoGen (Waltham, MA, USA). The drug was stored as a liquid at 4 °C, protected from light, and used before its expiration date, as recommended by the manufacturer. Rats were randomly assigned to Elahere or Vehicle groups. Animals in the Elahere group (n = 8) were treated with 20 mg/kg of Elahere (prepared to 2 mg/mL for administration) via intravenous (IV) tail vein injection once weekly for five weeks. The Vehicle group (n = 5) received an equivalent volume (10 mL/kg) of 5% glucose solution IV following the same schedule. Corneal tissues from the Vehicle group (5 rats, 10 eyes) were pooled into 2 biological replicate samples, and tissues from the Elahere group (8 rats, 16 eyes) were pooled into 3 biological replicate samples for scRNA-seq analysis.

### 4.2. Ocular Toxicity Assessment

Rats were monitored weekly during the study period for general health and specific ocular symptoms, including redness, discharge, and corneal opacity. Corneal integrity was evaluated every 7 days using fluorescein sodium staining. A 2 µL drop of 0.2% sodium fluorescein solution was instilled into the lower conjunctival sac, and the eye was gently closed for 2 s. Excess fluorescein was rinsed with sterile saline, and the corneal surface was examined under a slit-lamp microscope with a cobalt blue filter. Epithelial damage was visualized as areas of green fluorescence. At the study endpoint (week 5), rats were euthanized via CO_2_ asphyxiation followed by cervical dislocation. Selected eyes and optic nerves were collected, fixed in 10% neutral buffered formalin for at least 24 h, processed through graded alcohols, cleared in xylene, and embedded in paraffin wax. Sections (5 µm thick) were stained with hematoxylin and eosin (H&E) and examined by a board-certified veterinary pathologist blinded to treatment groups. Histopathological evaluation focused on corneal epithelial integrity, cellular morphology, inflammation, and pathological changes.

### 4.3. Corneal Tissue Processing and Single-Cell Sequencing

After euthanasia, the eyes were enucleated, and the corneas, including the limbal region, were carefully dissected in ice-cold phosphate-buffered saline (PBS). Single-cell suspensions were prepared via enzymatic digestion (dispase II, 2.4 U/mL; collagenase A, 1 mg/mL; Roche) and gentle mechanical dissociation in Hank’s Balanced Salt Solution at 37 °C for 30–60 min. Tissues were triturated with wide-bore pipette tips to release individual cells, and the suspensions were filtered through 40 µm cell strainers to remove debris. Cell viability was assessed using trypan blue exclusion staining, and cells were counted on an automated cell counter (Countess II, Thermo Fisher Scientific, Waltham, MA, USA). Viable cells (>80%) were adjusted to the input range for scRNA-seq. Single-cell transcriptomic profiling was performed using the BD Rhapsody™ HT Single-Cell Analysis System (BD Biosciences, San Jose, CA, USA), following the manufacturer’s protocol. Single cells were captured in microwells on BD Rhapsody™ cartridges. Messenger RNA (mRNA) from captured cells was reverse transcribed into complementary DNA and barcoded with unique molecular identifiers (UMIs) and cell labels. Libraries were prepared using the BD Rhapsody™ mRNA Whole Transcriptome Analysis (WTA) Kit, following the manufacturer’s protocol. Library quality was assessed using an Agilent Bioanalyzer and Qubit fluorometer. Sequencing was performed on an Illumina NovaSeq 6000 platform (Illumina, CA, USA), targeting a depth of ≥50,000 reads per cell with paired-end reads (2 × 75 bp or 2 × 100 bp).

### 4.4. Single-Cell RNA-Seq Data Processing

Raw sequencing reads were processed with the BD Rhapsody™ Analysis Pipeline software (v2.2.1) and aligned to the rat reference genome (GRCm39, GENCODE release M35). Analyses were performed in Python (v3.10.17) following the OmicVerse workflow [39]. Cells with fewer than 500 detected genes, fewer than 1000 UMIs, or mitochondrial gene content > 5% were excluded from the analysis. Doublets were removed using sccomposite (v1.0.0). Normalized data (shiftlog|pearson mode) were used to identify the top 2000 highly variable genes. Principal component analysis (PCA) was conducted and samples were integrated using Harmony. Uniform manifold approximation and projection (UMAP) was employed for two-dimensional visualization of the top 30 principal components (PCs). Cell clusters were annotated based on the following canonical marker genes and literature references: corneal epithelial cells: *Krt12*, *Krt15*, and *Krt14*; stromal cells (keratocytes): *Kera* and *Col1a1*; and endothelial cells: *Pecam1*. Immune cells were classified into subtypes using the following markers: myeloid cell: *Adgre1*, *Cd68*, and *Lyz1*; T cells: *Cd3d* and *Cd3e*; and natural killer (NK) cells: *Nkg7* and *Klrd1*. Annotations were refined using the top differentially expressed genes (DEGs).

### 4.5. Differential Gene Expression Analysis

Differentially expressed genes (DEGs) between the Elahere and Vehicle groups within each cell type were identified using the rank_genes_groups function in Scanpy (v1.10.4), which employs the Wilcoxon Rank-Sum test. This non-parametric test is robust for single-cell data. Genes were considered significant based on an adjusted *p*-value (Benjamini–Hochberg correction) of <0.05. To focus on genes with more substantial changes, an additional, secondary filter requiring a log2 fold-change > 0.25 was applied.

### 4.6. Pathway Enrichment Analysis

Gene Ontology pathway enrichment analyses were conducted on the DEG sets within key cell types to understand the broader biological functions and pathways affected by Elahere treatment. These analyses were conducted using the pygsea package [40], and a false discovery rate < 0.05 was considered significant. This step provided functional context to the DEGs.

### 4.7. Pseudotime Trajectory Analysis

Cellular differentiation trajectories for epithelial lineages were inferred using Monocle (v2.32.0) [41]. Normalized expression data from relevant cell subclusters were used for this analysis. Cell ordering along the trajectory was based on the highly variable or DEGs. Dimensionality reduction was performed using DDRTree, followed by the generation of a principal graph and the assignment of pseudotime values. The root state (pseudotime = 0) was defined as the limbal stem cell (LSC) cluster, expressing *Krt15* (the origin of epithelial differentiation). Gene expression trends along pseudotime were visualized using generalized additive models.

### 4.8. Cell–Cell Interaction Analysis

Intercellular communication was analyzed using CellChat (v2.1.2), leveraging the CellChatDB database with ortholog mapping for rat genes [42]. Significant ligand-receptor interactions between “sender” and “receiver” cell populations were inferred, and probabilities were adjusted for cell population size. Outputs included communication strength, significant ligand-receptor pairs, and active signaling pathways. Results were visualized as circle plots (network structure), heatmaps (pathway activity), and bubble or chord diagrams (specific interactions).

### 4.9. Statistical Analysis

Statistical analyses were performed using R (v4.4.2). Two groups were compared using unpaired Student’s *t*-tests for normally distributed data or Wilcoxon rank-sum tests for non-normal data. Multiple groups were compared using one-way ANOVA with post hoc tests. For proportional differences in cell types (e.g., in Figure 2D pie charts and bar plots), chi-square tests were used to assess significance. Data are expressed as mean ± standard errors of the mean or means ± standard deviations. A *p*-value < 0.05 was considered statistically significant.

## 5. Conclusions

This study aimed to identify cell-type-specific mechanisms of Elahere-induced ocular toxicity, dissect multi-compartmental (endothelial/immune/epithelial/stromal) cellular responses via scRNA-seq, and provide insights for mitigating its ocular side effects. Key findings include: (1) a shift in endothelial subtypes indicative of vascular injury; (2) a profound depletion of myeloid cells (from 9.05% to 1.31%); (3) an epithelial differentiation blockade characterized by a ~2.6-fold LSC expansion, leading to ineffective regeneration; and (4) pro-fibrotic stromal remodeling, marked by the severe loss of progenitor-like cells. This study provides an unprecedented high-resolution view of the cellular and molecular mechanisms underlying Elahere-induced corneal toxicity. The vulnerable cell populations and dysregulated pathways, including Folr1-mediated endothelial injury and FcγR-driven immune cell depletion, were identified, establishing a mechanistic framework for ADC-induced keratopathy. These findings pave the way for targeted therapeutic strategies to mitigate ocular toxicities and enhance the safety profile of ADCs, ultimately improving patient outcomes in oncology.

## Data Availability

The raw single-cell RNA sequencing datasets generated during the current study will be deposited in the Gene Expression Omnibus (GEO) repository.

## Supplementary Materials

The following supporting information can be downloaded at: https://www.mdpi.com/article/10.3390/ph18101492/s1, Supplementary Figure S1: Detailed Corneal Cell Type Annotation. Dot plot showing the top 3 marker genes for each of the 20 identified cell clusters. Supplementary Figure S2: Extended Analysis of Immune Cell Subsets. Violin plots showing additional DEGs or pathways in macrophage subpopulations beyond Figure 3E.

## Abbreviations

ADCs	Antibody-Drug Conjugates
ADCC	Antibody-Dependent Cellular Cytotoxicity
ADCP	Antibody-Dependent Cellular Phagocytosis
DEGs	Differential Expressed Genes
ECM	extracellular matrix
FcγR	Fc gamma receptor
FRα/Folr1	folate receptor α
GEO	Gene Expression Omnibus
H&E	Hematoxylin and Eosin
IACUC	Institutional Animal Care and Use Committee
LSC	limbal stem cell
MHC	Major Histocompatibility Complex
NK	natural killer
PCA	Principal Component Analysis
scRNA-seq	single-cell RNA sequencing
TGFβ	Transforming Growth Factor Beta
UMAP	Uniform Manifold Approximation and Projection
UMIs	unique molecular identifiers
VEGF	Vascular Endothelial Growth Factor
