# Supplementary material for: Single-Cell Transcriptomics Reveals a Multi-Compartmental Cellular Cascade Underlying Elahere-Induced Ocular Toxicity in Rats
Source: Pharmaceuticals (Basel). 2025 Oct 4;18(10):1492. doi: 10.3390/ph18101492 (PMC12567386; doi:10.3390/ph18101492)
Supplement: Supplementary file 1 [file pharmaceuticals-18-01492-s001.zip › pharmaceuticals-3852443-supplementary.pdf]

Supplementary Figures

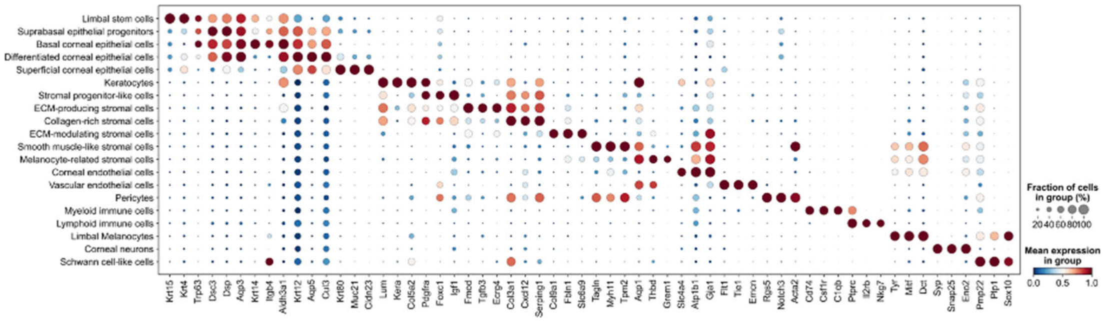

Supplementary Figure S1: Detailed Corneal Cell Type Annotation. Dot plot showing the top 3 marker genes for each of the 20 identified cell clusters.

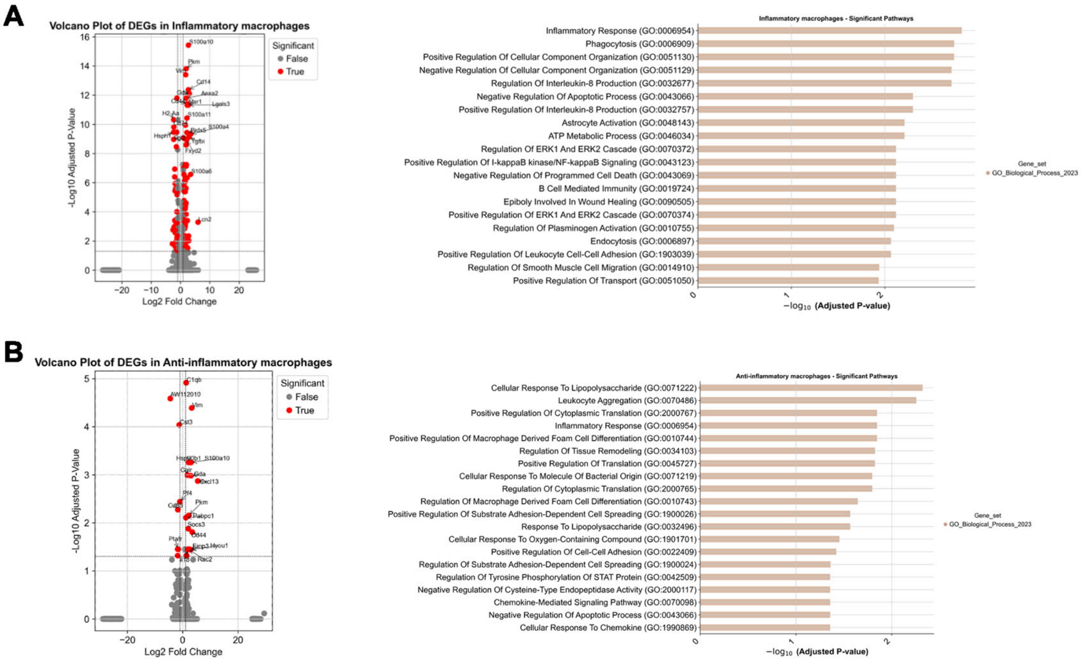

Supplementary Figure S2: Extended Analysis of Immune Cell Subsets. Violin plots showing additional DEGs or pathways in macrophage subpopulations beyond Figure 3E.
